# Isoliquiritigenin Induces Mitochondrial Dysfunction and Apoptosis by Inhibiting mitoNEET in a Reactive Oxygen Species-Dependent Manner in A375 Human Melanoma Cells

**DOI:** 10.1155/2019/9817576

**Published:** 2019-01-21

**Authors:** Xiao-Yu Chen, Huan-Huan Ren, Dan Wang, Ying Chen, Chuan-Jun Qu, Zhao-Hai Pan, Xiao-Na Liu, Wen-Jin Hao, Wen-Juan Xu, Ke-Jun Wang, De-Fang Li, Qiu-Sheng Zheng

**Affiliations:** ^1^School of Integrated Traditional Chinese and Western Medicine, Binzhou Medical University, Yantai, 264003 Shandong, China; ^2^Key Laboratory of Xinjiang Endemic Phytomedicine Resources of Ministry of Education, School of Pharmacy, Shihezi University, Shihezi, 832002 Xinjiang, China

## Abstract

The mitochondrial protein mitoNEET is a type of iron-sulfur protein localized to the outer membrane of mitochondria and is involved in a variety of human pathologies including cystic fibrosis, diabetes, muscle atrophy, and neurodegeneration. In the current study, we found that isoliquiritigenin (ISL), one of the components of the root of *Glycyrrhiza glabra L.*, could decrease the expression of mitoNEET in A375 melanoma cells. We also demonstrated that mitoNEET could regulate the content of reactive oxygen species (ROS), by showing that the ISL-mediated increase in the cellular ROS content could be mitigated by the mitoNEET overexpression. We also confirmed the important role of ROS in ISL-treated A375 cells. The increased apoptosis rate and the decreased mitochondrial membrane potential were mitigated by the overexpression of mitoNEET in A375 cells. These findings indicated that ISL could decrease the expression of mitoNEET, which regulated ROS content and subsequently induced mitochondrial dysfunction and apoptosis in A375 cells. Our findings also highlight mitoNEET as a promising mitochondrial target for cancer therapy.

## 1. Introduction

Melanoma is a malignancy of pigment-producing melanocytes that is diagnosed in approximately 132,000 individuals globally every year [1]. The diagnosis of melanoma is associated with a health burden and economic loss due to age-dependent incidence, primarily affecting individuals between the ages of 30 and 50. Indications for radiotherapy as a therapeutic approach are limited because melanoma is considered to be radioresistant traditionally [2]. In preclinical studies, a solid rationale could explain poor clinical outcomes of traditional radiotherapy. Sensitivity to conventional fractionation (1.8–3 Gy) is low in vitro in melanoma cells [3]. Current treatments for stage IV melanoma patients are limited and have shown little improvement. Therefore, alternative and effective therapies are sorely needed for a potential cure of this aggressive cancer.

Apoptosis, as one of the most clearly characterized processes of cell death, plays an important role in eliminating damaged cells. Cell apoptosis has obvious morphological and biochemical changes, such as DNA division into 180–200 base pairs, cellular fragmentation into apoptotic bodies, and phosphatidylserine externalization [4]. Caspase-3 activation in apoptotic cells cleaves poly (ADP-ribose) polymerase (PARP) into two fragments sized 31 and 85 kD; the resultant PARP inactivation leads to nucleosomal DNA fragmentation. There are two main apoptotic pathways: intrinsic (mitochondrial) and extrinsic (death receptor) [5]. The pivotal role of the mitochondria in apoptosis has been widely demonstrated [6, 7]. During apoptosis, mitochondria release soluble proteins, including cytochrome c and Smac/DIABLO, from the intermembrane space to initiate the activation of caspases in the cytosol [8, 9]; the release of these proteins is a consequence of the compromised integrity of the mitochondrial outer membrane through its permeabilization. The dynamic events occurring within the mitochondria ultimately determine the initiation of apoptosis, emphasizing the close relationship between mitochondrial dysfunction and cell death.

Reactive oxygen species (ROS) are typically small, short-lived, highly active molecules [10, 11]. The first ROS-producing mitochondrial site is complex III located in the mitochondrial inner membrane [12]. Mitochondrial ROS production occurs mainly on the electron transport chain (ETC) [13, 14]. At physiological levels, ROS acts as a redox messenger in intracellular signal transduction and regulation, whereas excess cell death was induced by ROS via the intrinsic apoptotic pathway. Mitochondrial DNA (mtDNA) damage is a common event in the mitochondrial apoptotic pathway. Mitochondrial DNA damage impairs the transcription of mitochondrial proteins involved in ETC, leading to the rupture of respiratory chain, further increasing ROS production, and ultimately the loss of mitochondrial membrane potential (MMP) and damage to ATP synthesis [15]. ROS can oxidize phospholipid cardiolipin which binds cytochrome c to the outer lobe of the inner mitochondrial membrane [16]. Normally, cytochrome c shuttles electrons between the mitochondrial complexes III and IV. ROS-oxidized cardiolipin reduces the binding of cytochrome c and increases the level of free cytochrome c, which is likely to be released into the cytoplasm through the mitochondrial outer membrane and initiates apoptotic cascade (Circu et al., 2010). Thus, ROS can trigger mitochondrial dysfunction and apoptosis.

The mitochondrial protein mitoNEET (also known as CDGSH iron-sulfur domain 1 (CISD1)) is a member of iron-sulfur protein and is involved in a variety of human pathologies including diabetes, Wolfram syndrome 2, cystic fibrosis, muscle atrophy, and neurodegeneration. [17–19]. Early studies have shown that mitoNEET plays a key role in regulating cellular energy use and lipid metabolism. [20]. mitoNEET-null mice appeared to have a mitochondrial dysfunction [21], whereas mice overexpressing mitoNEET gained weight by increasing fat tissue [22]. Sohn and colleagues found that mitoNEET played a vital role in breast cancer cell proliferation and the formation of tumor [23]. Furthermore, the overexpression of mitoNEET in the MDA-MB-231 breast cancer cell line led to an increase in mitochondrial ETC complexes and tumor growth [23]. Collectively, mitoNEET may not only regulate energy consumption but also function as a novel antitumor therapeutic target. Recent studies on the effects of redox regulation by mitoNEET demonstrated that mice overexpressing mitoNEET exhibited decreased ROS generation from the mitochondria; however, oxidative phosphorylation and electron transport were significantly upregulated in the absence of mitoNEET [21, 23], suggesting that mitoNEET may also regulate the generation of ROS.

Isoliquiritigenin (ISL), one of the flavonoids of the root of *Glycyrrhiza glabra L.*, was previously shown to have antioxidant, anti-inflammatory, and tumor-suppressive effects [24–26]. Previously, we demonstrated that ISL could induce apoptosis in HeLa cells by inducing intracellular ROS levels [27]. ISL inhibited proliferation and induced apoptosis by alleviating hypoxia and reducing glycolysis in B16F10 mouse melanoma cells [28]. In this first study to provide novel insights into the therapeutic targets of ISL and elucidate the mechanism of ISL-induced mitochondrial dysfunction and apoptosis in melanoma cells, we hypothesized that mitoNEET might influence the proliferation and ROS regulation in ISL-treated A375 melanoma cells. We therefore examined the role of mitoNEET in ISL-induced mitochondrial dysfunction and apoptosis in A375 cells.

## 2. Material and Methods

### 2.1. Cell Culture

The A375 melanoma cell line was purchased from Shanghai Biological Institute (Shanghai, China), and cultures were maintained in Dulbecco's modified Eagle medium supplemented with 10% fetal bovine serum, penicillin/streptomycin (1 : 100), and 4 mM L-glutamine in a CO_2_ incubator at 37°C.

### 2.2. Reagents

ISL (purity ≥ 98%) was purchased from Jiangxi Herb Tiangong Technology (Jiangxi, China). 2,7-Dichlorodihydro-fluorescein diacetate (DCFH-DA), dimethyl sulfoxide (DMSO), 3-(4,5-dimethylthiazol-2-yl)-2,5-diphenyl tetrazolium bromide (MTT), N-acetylcysteine (NAC), and L-buthionine sulfoximine (BSO) were purchased from Sigma. MitoSOX Red was purchased from Invitrogen (Carlsbad, CA, USA). 2,7-Dichlorodihydro-fluorescein diacetate (DCFH-DA), MitoTracker Green, JC-1, and apoptosis detection kits were purchased from Kaiji Biotech (Nanjing, China). Hoechst 33258, RIPA buffer, and GSH/GSSG detection kit were purchased from Beyotime Biotech (Shanghai, China). RNA isolation, cDNA synthesis, and SYBR Premix kits were purchased from Takara Biomedical Technology (Beijing, China). Membrane Protein Isolation Kit was purchased from Invent Biotechnologies (USA). All antibodies were obtained from Cell Signaling Technology (Cell Signaling, USA).

### 2.3. Overexpression of mitoNEET

mitoNEET cDNA was amplified by reverse transcription- (RT-) polymerase chain reaction (PCR) from the total RNA of A375 cells with primers and cloned into a pLVX-CMV-MCS-T2A-Zsgreen plasmid (details of cloning provided in Supplemental Data (available here)). A375 cells were transfected with the empty vector or the mitoNEET vector using Lipofectamine 2000 (Invitrogen), according to the manufacturer's instructions. The cells were collected 48 h after transfection, and cell lysates were immunoblotted for the indicated proteins.

### 2.4. Cell Proliferation Inhibition Assay

The effect of ISL on A375 cell proliferation was determined by measuring the absorbance of MTT. Cells (5 × 10^4^ per well) were seeded in 96-well microtiter plates and treated with indicated ISL doses for 24 h. Next, 20 *μ*L MTT solution (5 mg/mL in phosphate-buffered saline) was added to each well for an additional 4 h at 37°C. The MTT solution with the medium was discarded, and 200 *μ*L dimethyl sulfoxide was added to each well to dissolve the formazan crystals. Absorbance was measured at 570 nm using a Tecan Infinite M200 microplate reader (Tecan, USA).

### 2.5. Hoechst 33258 Staining

Hoechst 33258 staining was used to detect apoptotic morphological changes in nuclear chromatin. A375 cells were seeded in six-well plates and treated with ISL after 24 h. After pretreatment, the medium was removed and cells were fixed with 4% paraformaldehyde for 10 min at room temperature. After two washes with PBS, the cells were incubated with 0.5 mL Hoechst 33258 staining solution for 5 min. The morphological shape of the nucleus was viewed under an Olympus IX-70 fluorescence microscope (Olympus, Tokyo, Japan).

### 2.6. Flow Cytometry

Flow cytometric determination of apoptosis, number of mitochondria, MMP, and mitochondrial superoxide, and total ROS levels was performed using a FACSCalibur flow cytometer (BD Biosciences, Mountain View, CA, USA) and analyzed using the CellQuest software (BD Biosciences). The rate of apoptosis was determined using the annexin V/propidium iodide (PI) double staining method. The mitochondrial number, MMP, mitochondrial superoxide level, and intracellular ROS level were determined by incubating the cells with 100 nM MitoTracker Green for 15 min, 1 mM JC-1 for 30 min, 5 *μ*M MitoSox Red for 20 min, and 10 mM DCF-DA for 20 min at 37°C before flow cytometric analyses, before analysis by flow cytometry.

### 2.7. Assessment of Cellular Morphology by Confocal Laser Scanning Microscopy

The previously described method by Quoilin et al. [29] was used to observe the morphological changes in A375 cells. A phase-contrast microscope equipped with a digital camera (Axio Observer, Zeiss, Germany) was used to observe and record the morphological changes of A375 cells after 24 h incubation with different concentrations of ISL. Specific fluorescent probes were used to detect mitochondrial activity and cellular oxidative stress including MitoTracker Green (100 nM for 20 min) for the mitochondrial number (Cottet-Rousselle et al., 2011), the potentiometric fluorescent dye JC-1 (1 mM for 10 min) for MMP, and MitoSOX Red (5 *μ*M for 20 min) and DCF (10 mM for 10 min) for mitochondrial and total cellular ROS in living cells, respectively (Cottet-Rousselle et al., 2011; Mukhopadhyay et al., 2007). A375 cells were cultured on glass coverslips in 6-well plates and incubated at 37°C with the specific dyes before image capturing.

### 2.8. Measurement of Cytochrome c Release

A double antibody sandwich enzyme-linked immunosorbent assay kit (Enzo Life Sciences, Belgium) was used to detect cytosolic and mitochondrial cytochrome c concentrations, as described previously by Quoilin et al. [29]. Briefly, A375 cells were treated with different concentrations of ISL for 6 h. Cells were digested and suspended in PBS at a final concentration of 10^7^ cells/mL. Next, the cell suspension was centrifuged at 500 *g* for 5 min. After remove supernatant, digitonin cell permeabilization buffer was added. The pellet was incubated with digitonin cell permeabilization buffer for 5 min on ice, then centrifuged at 100 *g* at 4°C for 10 min. The supernatant which contains the cytosolic fraction with cytochrome c (fraction I) was collected and kept on ice. RIPA cell lysis buffer was used to dissolve the pellet. After 15 min incubation on ice, the lysate was centrifuged at 5000 *g* at 4°C for 10 min. The supernatant which contains the mitochondrial fraction with cytochrome c (fraction II) was collected and kept on ice. According to the ELISA kit protocol, aliquots from fractions I and II were pipetted into wells of the 96-well plate, followed by the addition of appropriate antibodies, conjugates, and substrates into each well. The absorbance was detected at 405 nm by a Tecan Infinite M200 microplate reader.

### 2.9. Measurement of Complex I, II, III, and IV Activity Levels

Complex I and IV activity levels were measured by a commercial kit (Genmed, USA) following the manufacturer's instructions. Complex II and III activity levels were measured by a commercial kit from Cayman (USA).

### 2.10. GSH/GSSG Ratio

Ultrasonication was used to prepare cell extracts. Cell extracts in ice-cold 5% metaphosphoric acid was centrifuged at 10,000 *g* for 20 min, and the supernatants were collected. The GSH content and T-GSH/GSSG of the supernatants were, respectively, determined by commercial kits (NJBC, Nanjing, China). The absorbance at 420 nm was measured using a spectrophotometer. Reduced GSH levels were determined by subtracting the 2 × GSSG values from the T-GSH values, and the GSH/GSSG ratio was calculated.

### 2.11. RNA Isolation and Relative Quantitative Real-Time RT-PCR

Total RNA was extracted from A375 cells using RNAiso Plus (Takara) and stored at −80°C until further use. cDNA was synthesized from total RNA with a PrimeScript RT reagent kit (Takara). PCR reaction was performed using the SYBR Premix Ex Taq II (Takara) in a Lightcycler 480 (Roche). The results were normalized based on glyceraldehyde 3-phosphate dehydrogenase (GAPDH) expression, and the 2^−∆∆*CT*^ method was used to analyze the relative levels of mRNA (Schmittgen et al., 2008). The primer sequences were as follows (5′-3′): mitoNEET, forward CGA GTT GAA TGG ATC GCA GC, reverse ACA ACG GCA GTA CAC AGC TT; *β*-actin, forward AGA AAA TCT GGC ACC ACA CC, reverse TAG CAC AGC CTG GAT AGC AA.

### 2.12. Western Blotting

A375 cells were lysed with RIPA buffer (Beyotime) supplemented with protease inhibitors (Beyotime). Mitochondrial membrane proteins were extracted using the mitochondrial membrane protein isolation kit (Invent Biotechnologies). The lysates were centrifuged at 12,000 *g* for 10 min at 4°C, and the protein concentrations were determined by a BCA Protein Assay Kit. Then the protein samples were denatured at 100°C for 10 min. Equal amounts of protein were loaded in each well of 10% sodium dodecyl sulfate polyacrylamide gels and transferred to polyvinylidene fluoride membranes (Millipore, Billerica, MA, USA), blocked with 5% nonfat milk for 1 h at room temperature, and then incubated with antibodies specific for mitoNEET, cleaved PARP, cleaved caspase-3, and tubulin (Cell Signaling, USA) at 4°C overnight. Signals were detected with horseradish peroxidase-conjugated secondary antibodies using a chemiluminescence process (Millipore) as per the manufacturer's instructions. Protein bands were detected on a bioimaging system (Bio-Rad, Berkeley, CA, United States).

### 2.13. Statistical Analysis

Data were expressed as the means ± standard deviation (SD). Statistical differences were analyzed by one-way analysis of variance followed by multiple comparisons performed with the Bonferroni post hoc test (SPSS version 18.0). *P* values < 0.05 were considered statistically significant.

## 3. Results

### 3.1. ISL Inhibits A375 Cell Proliferation and Induces Apoptosis

**Figure 1:**
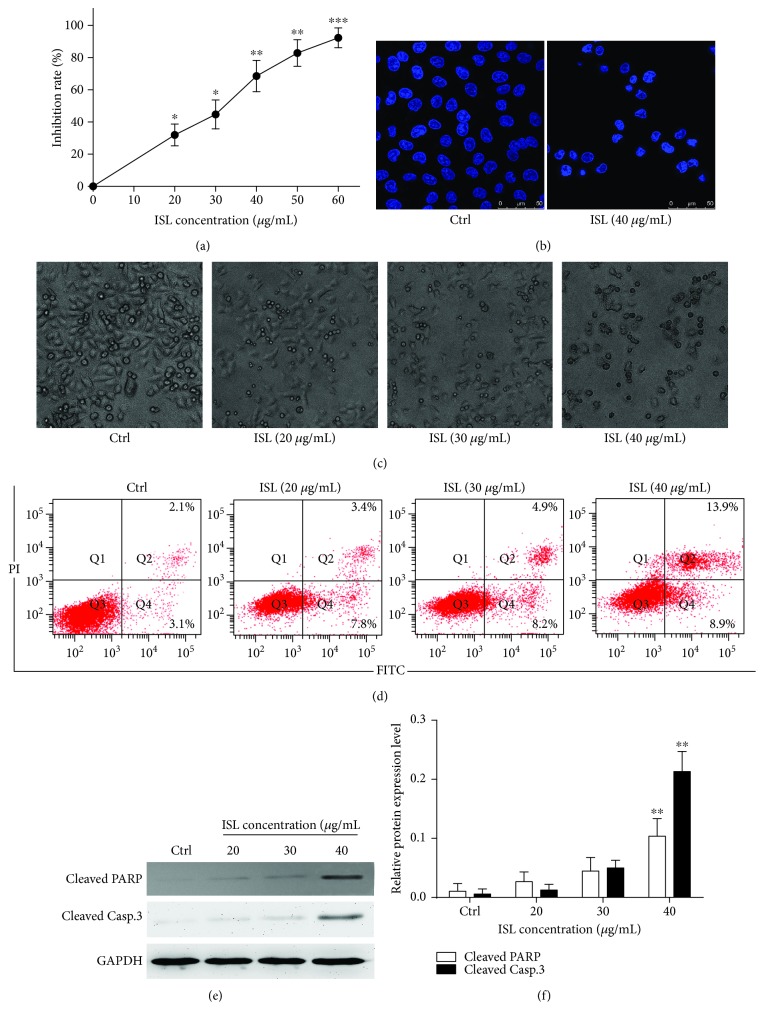
Isoliquiritigenin induces apoptosis in A375 melanoma cells. (a) Cell proliferation rate was detected by the MTT assay. (b) Hoechst 33258 staining showing nuclear shrinkage in A375 cells. (c) Phase-contrast micrographs (200x) showing morphological changes in isoliquiritigenin- (ISL-) treated A375 cells. (d) Apoptosis was measured by flow cytometry using double stain with annexin V and propidium iodide in A375 cells treated with ISL. (e) Western blotting analysis for cleaved PARP and cleaved caspase-3. (f) Analysis grayscale of band for western blotting. Data are expressed as band intensities of target protein/band intensity of the housekeeping protein. ^∗^ *P* < 0.05 and ^∗∗^ *P* < 0.01 versus control.

ISL inhibited the proliferation of A375 cells in a dose-dependent manner (Figure 1(a)). Specifically, treatment with ISL at 40 and 60 *μ*g/mL led to 69.86% and 92.22% reduction in the proliferation of A375 cells, respectively. As shown in Figure 1(b), staining with Hoechst 33258 revealed the presence of nuclear pyknosis in ISL-treated A375 cells. Additionally, the control cells exhibited a fusiform shape and higher transmittance. However, the cells treated by 40 *μ*g/mL ISL showed a shrunken cellular profile and lower transmittance compared with the control cells (Figure 1(c)). To quantify the rate of apoptosis, the cells were stained with FITC-conjugated annexin V and PI. Whereas the percentage of apoptotic cells was negligible in the control cultures, the 24 h ISL exposure led to a dose-dependent increase in both early and late apoptotic cells (Figure 1(d)). Furthermore, the protein levels of cleaved PARP and cleaved caspase-3 cells were significantly elevated after the ISL treatment (Figures 1(e) and 1(f)).

### 3.2. ISL Induces Mitochondrial Dysfunction in A375 Cells

**Figure 2:**
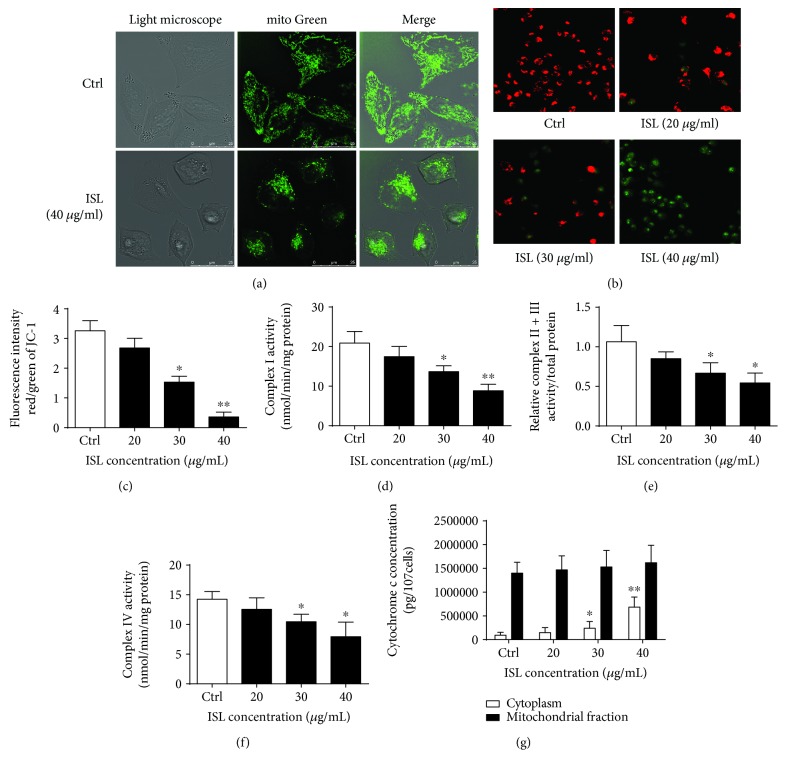
ISL induces mitochondrial dysfunction in A375 melanoma cells. (a) Representative confocal images showing the fluorescent distribution of MitoTracker Green (excitation, 495 nm; emission, 535 nm) in A375 cells with or without ISL treatment. (b) Representative confocal images showing the fluorescent JC-1 probe in A375 cells treated with different concentrations of ISL. (c) Quantification of the JC-1 fluorescence intensity detected by flow cytometry. (d–f) Complex I–IV activity analysis in ISL-treated A375 cells. (g) Cytosolic and mitochondrial cytochrome c concentrations determined by enzyme-linked immunosorbent assay. ^∗^ *P* < 0.05 and ^∗∗^ *P* < 0.01 versus control.

MitoTracker Green staining showed that the mitochondria of the A375 cells treated by ISL formed an ovoid and multibranch-structured network (Figure 2(a)). Additionally, the JC-1 staining revealed that the MMP decreased following ISL treatment (Figures 2(b) and 2(c)). In parallel, the activity levels of complexes I–IV were reduced with ISL treatment (Figures 2(d)–2(f)). We also determined the levels of cytosol cytochrome c and mitochondria cytochrome c in A375 cells by ELISA, which revealed that the cytosolic cytochrome c levels were significantly increased after 24 h of ISL treatment; however, no significant changes were observed in the mitochondrial cytochrome c content (Figure 2(g)).

### 3.3. ISL Triggers ROS Generation, Which Contributes to ISL-Induced Apoptosis and Mitochondrial Dysfunction in A375 Cells

**Figure 3:**
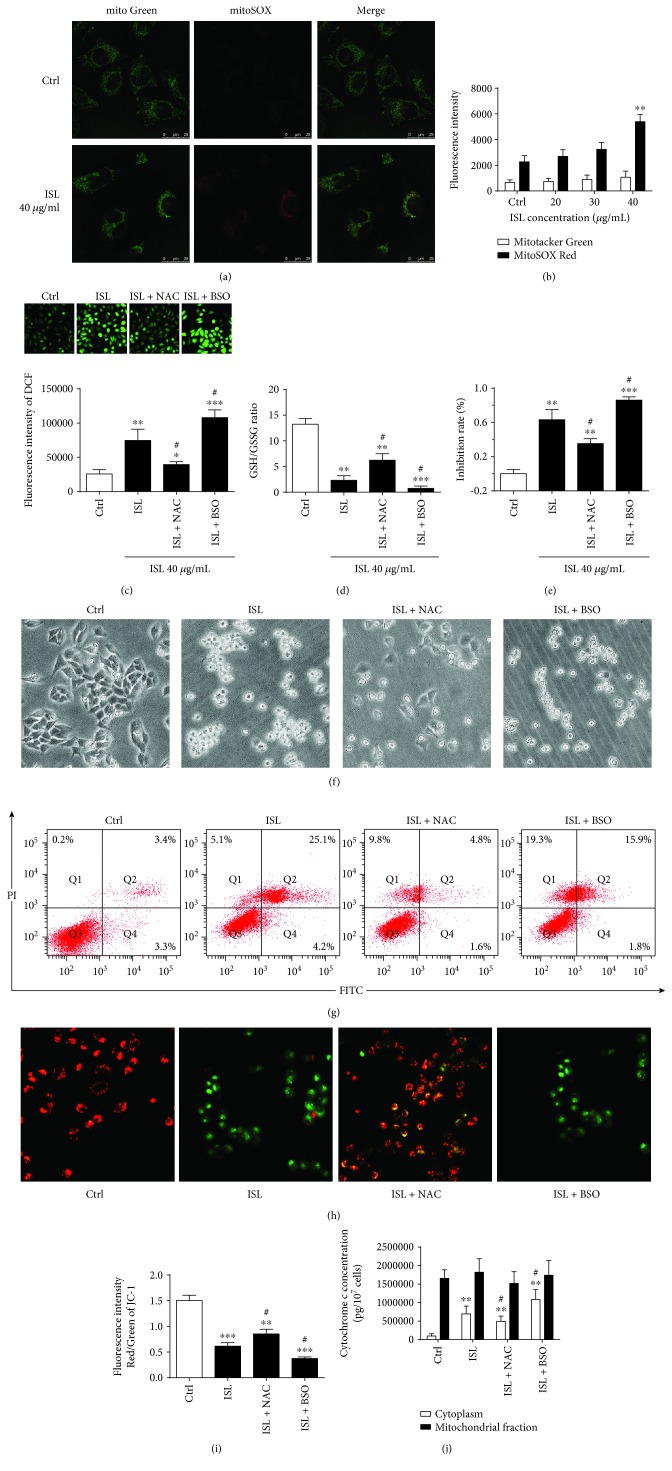
ISL triggers the production of reactive oxygen species, which contributes to ISL-induced apoptosis and mitochondrial dysfunction in A375 cells. (a) Representative confocal microscopy images of mitochondrial network in A375 cells treated with or without ISL. (b) Quantification of the MitoSOX Red and MitoTracker Green fluorescence intensity detected by flow cytometry. (c) Representative confocal microscopy images of DCFH-DA and quantification of the DCFH-DA fluorescence intensity by flow cytometry. (d) Analysis of the GSSH/GSH ratio. (e) The inhibition rates of A375 cells treated by ISL alone or ISL cotreated with NAC or BSO. (f) Phase-contrast micrographs (200x) showing morphological changes in A375 cells treated with ISL alone or ISL cotreated with NAC or BSO. (g) Apoptosis rates of A375 cells determined by annexin V/PI double staining in response to indicated treatments. (h) Representative confocal microscopy images of the JC-1 probe in A375 cells treated by ISL alone or ISL cotreated with NAC or BSO. (i) Quantification of the JC-1 fluorescence intensity in A375 cells by flow cytometry. (j) Cytosolic and mitochondrial cytochrome c concentrations in A375 cells treated by ISL alone or ISL cotreated with NAC or BSO. ^∗^ *P* < 0.05, ^∗∗^ *P* < 0.01, and ^∗∗∗^ *P* < 0.001 versus control; ^#^ *P* < 0.05 versus ISL.

To assess the effect of ISL on mitochondrial ROS generation, we loaded A375 cells with MitoSOX Red which is a fluorescent probe which specifically targets mitochondria ROS in viable cells. We found that MitoSOX Red exhibited a near-complete colocalization with the MitoTracker Green probe (yellow areas) and that the fluorescence intensity of MitoSOX Red was significantly increased in cultures treated with 40 *μ*g/mL ISL compared with the control cultures (Figure 3(a)). As shown in Figure 3(b), the fluorescence intensity of MitoSOX Red, as detected by flow cytometry, was increased 2.4-fold in cultures treated with 40 *μ*g/mL ISL for 6 h compared with the control cells (Figure 3(b)).

We next utilized DCFH-DA to fluorescently detect total cellular ROS levels. As shown in Figure 2(c), the untreated cells showed a slight green DCFH-DA fluorescence, indicating the presence of low-level ROS in the cells. In contrast, the ISL-treated cells showed an apparent green fluorescence, indicating that ISL could induce additional ROS production in A375 cells. The histogram showed that the intracellular ROS levels were increased 3.2-fold in cultures that were treated with ISL for 6 h compared with the control cultures (Figure 2(c)). Cotreatment of the cultures with NAC and ISL led to a significant reduction in the total ROS levels; however, the levels of total ROS were significantly increased in cultures cotreated with ISL and BSO (Figure 2(c)).

The GSH/GSSG ratio was significantly decreased in A375 cells treated with ISL and significantly increased in cultures cotreated with ISL and NAC. Additionally, the reduction in the GSH/GSSG ratio was worse in cultures cotreated with ISL and BSO than in cultures treated with ISL alone (Figure 3(d)). Furthermore, the inhibition rate was lower in cultures cotreated with ISL and NAC than those treated with 40 *μ*g/mL ISL group, indicating a protective effect. The inhibition rate was significantly higher compared with the cultures cotreated with ISL and BSO (Figure 3(e)).

As shown in the representative images in Figure 3(f), we also evaluated the changes in a cell shape in response to ISL treatment. The control cells exhibited a fusiform shape, whereas the ISL-treated cells were smaller in size with lower transmittance, indicating that the overall state of the ISL-treated cells was worse with compromised attachment. In contrast, stable attachment to the culture dish surface was observed in cultures treated with NAC, whereas cotreatment with ISL and BSO led to the reduction in the size of cells which exhibited compromised attachment ability (Figure 3(f)).

Both the early and late apoptosis rates were decreased by NAC and ISL cotreatment of the A375 cells. As seen in Figure 3(g), 19.3% of the cells were annexin V-negative and PI-positive, indicating that ISL cotreatment with BSO might cause necrosis in A375 cells (Figure 3(g)). The MMP was also higher in cells cotreated with ISL and NAC compared with ISL-treated cells, which was reduced in cells cotreated with ISL and BSO (Figures 3(h) and 3(i)). The cytosolic cytochrome c levels were also significantly decreased by ISL cotreatment with NAC, which were increased in cells cotreated with ISL and BSO. However, no significant changes in mitochondrial cytochrome c content were observed (Figure 3(j)).

### 3.4. ISL Inhibits the Expression of mitoNEET, Which Regulates ROS

**Figure 4:**
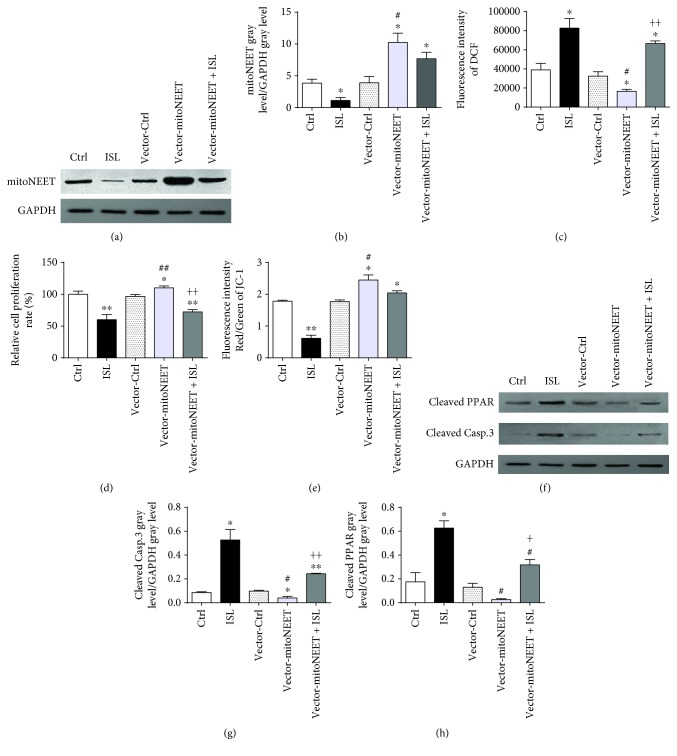
ISL inhibits the expression of mitoNEET, which regulates ROS, and subsequently induces apoptosis and mitochondrial dysfunction in A375 cells. (a) ISL inhibits the protein expression of mitoNEET in A375 cells detected by western blotting. (b) Relative band intensities of mitoNEET in empty vector- or mitoNEET-overexpressing cells with or without ISL treatment. (c) ISL regulates cellular ROS levels by controlling the mitoNEET expression. (d) ISL induces proliferation inhibition of A375 cells by inhibiting the expression of mitoNEET. (e) ISL decreases mitochondrial membrane potential by inhibiting the expression of mitoNEET in A375 cells. (f) ISL increases the expression of cleaved PARP and cleaved caspase-3 by inhibiting the expression of mitoNEET. (g–h) Relative band intensities of cleaved PARP and cleaved caspase-3 in empty vector- or mitoNEET-overexpressing cells with or without ISL treatment. ^∗^ *P* < 0.05 and ^∗∗^ *P* < 0.01 versus control; ^#^ *P* < 0.05 and ^##^ *P* < 0.01 versus ISL; and ^+^ *P* < 0.05 and ^++^ *P* < 0.01 versus vector-Ctrl.

To determine whether ISL impacted the expression of mitoNEET, we first determined the protein expression levels of mitoNEET by western blotting in A375 cells. As shown in Figure 4(a) and quantified in Figure 4(b), a significant decrease in mitoNEET expression was observed after ISL treatment. To confirm the effect of mitoNEET in ISL-treated A375 cells, we engineered an A375 cell line that overexpressed mitoNEET and confirmed that they exhibited a significant increase in the mitoNEET expression compared with the control cells that expressed the empty vector (vector-Ctrl cells). Intriguingly, ISL treatment of the mitoNEET-overexpressing cells led to a reduction in mitoNEET expression compared with the untreated mitoNEET-overexpressing cells (Figures 4(a) and 4(b)).

We next examined the consequence of mitoNEET overexpression on cellular ROS generation. The mitoNEET-overexpressing cells showed a significantly lower levels of cellular ROS compared with the vector-Ctrl cells. In addition, ISL treatment resulted in a significant increase in the level of ROS in the mitoNEET-overexpressing cells (Figure 4(c)). The proliferation rate of the mitoNEET-overexpressing cells was significantly higher than that of the vector-Ctrl cells, and ISL treatment of the mitoNEET-overexpressing cells led to the proliferation rate significant decrease (Figure 4(d)). Furthermore, the mitoNEET-overexpressing cells exhibited a significantly higher MMP compared with the vector-Ctrl cells. However, ISL treatment led to a significant decrease of MMP in the mitoNEET-overexpressing A375 cells (Figure 4(e)). The levels of cleaved PARP and cleaved caspase-3, which were significantly higher in the ISL-treated A375 cells compared with the control cells, were significantly lower in the ISL-treated mitoNEET-overexpressed cells (Figures 4(f)–4(h)). The expressions of cleaved PARP and cleaved caspase-3 were lower in the ISL-treated vector-mitoNEET cells compared with those in the ISL-treated cells, suggesting that mitoNEET might act as an antiapoptotic factor (Figures 4(f)–4(h)).

## 4. Discussion

Melanoma is the most aggressive form of skin cancer, and its poor prognosis is largely due to resistance to conventional chemotherapy with cytotoxic drugs. Compounds used in traditional Chinese medicine represent the characteristics of small molecules with weak receptor binding [30, 31], implying that they can bind not just one target receptor but many, exerting various effects. In contrast, the potential for severe toxicity and serious side effects is higher with chemical drugs that bind a single target with a strong affinity. In general, compounds used in traditional Chinese medicine have better effects compared with chemotherapeutic drugs in melanoma therapy.

ISL is an important medicinal derived from licorice [32] that was demonstrated to induce apoptosis through distinct molecular mechanisms in several cell lines [33–35]. However, ISL as an inducer of mitochondrial dysfunction was rarely reported. In this study, we found that ISL-induced apoptosis and mitochondrial dysfunction were associated with the decrease of mitoNEET expression, which regulates cellular ROS levels. We found that the rate of apoptosis was increased, with cytochrome c release from the mitochondria after ISL treatment in A375 cells. Apoptotic morphological changes including clear nuclear pyknosis were also observed. Moreover, the levels of cleaved PARP and cleaved caspase-3 were significantly increased by ISL treatment. Caspase-3 activation leads to the DNA repair enzyme PARP cleavage. Cleaved PARP, which occurs early during the execution phase of apoptosis, is considered a general hallmark of apoptosis [36]. So the increase of cleaved PARP and cleaved caspase-3 indicated ISL could induce A375 cell apoptosis. The mitochondrion is involved in several levels of the fate of a cell, whether it will survive or not, and plays a key role in cell apoptosis signaling by controlling cellular energy metabolism and contributing to the control of ROS levels [37]. We found that the mitochondrial distribution was organized, with an ovoid-shaped and a multibranch structure, in A375 cells treated by ISL. We also found that there was a sharp decline in the MMP following the ISL treatment. Mitochondrial respiratory chain complex I-IV activity was also simultaneously decreased by the ISL treatment. Finally, we determined that the cytosolic cytochrome c levels were significantly increased after the ISL treatment, in the absence of a significant change in the mitochondrial cytochrome c content.

Previous studies demonstrated that ISL could induce ROS generation [25, 27, 38]. We indeed observed that the mitochondrial ROS levels were increased in response to ISL in A375 cells. Additionally, we also showed that the levels of total cellular ROS were also increased by the ISL treatment. We utilized the ROS scavenger NAC and the ROS agonist BSO to further elucidate the effect of ROS on ISL-induced apoptosis and mitochondrial dysfunction in A375 cells and found that NAC was protective against ISL-induced apoptosis and mitochondrial dysfunction. We further found that NAC cotreatment with ISL led to the inhibition and reduction in the apoptosis rate in A375 cells. The cellular morphology also exhibited an obvious improvement compared with that observed in the ISL-treated cells. The cotreatment with NAC also led to an increase in the MMP and a decrease in the cytosolic cytochrome c levels. In contrast, cotreatment of the cells with ISL and BSO led to increased inhibition and apoptosis rates, and the necrosis of A375 cells could be clearly observed by annexin V/PI double staining. The decrease in MMP and the increase in cytosolic cytochrome c levels were also observed in A375 cells cotreated with ISL and BSO. Overall, these findings demonstrate that ROS play an important role in ISL-induced apoptosis and mitochondrial dysfunction in A375 cells.

Several studies have showed the effects of mitoNEET on disease associated with oxidative stress such as cancer, obesity, and Parkinson's disease [22, 23]. Human NEET family members, including CISD1, CISD2 (also known as NAF1), and CISD3 (also known as Miner2), are important stress response proteins involved in the regulation of iron and ROS accumulation in the mitochondria [39]. Previous studies showed that the overexpression of mitoNEET in MDA-MB-231 breast cancer cells led to an increase in the expression of mitochondrial ETC complexes as well as the proliferation of tumor [23]. L929 fibrosarcoma cells constitutively express high levels of mitoNEET, which is required for tumor necrosis factor alpha- (TNF-*α*-) induced necroptosis in the presence of caspase inhibition [40]. Furthermore, mitoNEET serves as a mitochondrial binding site for the TNF-*α*-induced translocation of the Stat3-Grim-19 complex. Suppression of the mitoNEET expression prevented the TNF-*α*-induced translocation of the Stat3-Grim-19 complex to the mitochondria, with suppression of Grim-19 or Stat3 expression preserving cell viability in hepatocytes exposed to ethanol and fructose and treated with TNF-*α* [39]. We found that the expression of mitoNEET was decreased by ISL treatment in A375 cells. We also found that the cellular ROS levels could be regulated by mitoNEET, as observed by the significant increase in ROS levels by the inhibition of mitoNEET expression by ISL. However, mitoNEET overexpression led to a reduction in the cellular ROS levels. Further analysis revealed that the mitoNEET overexpression led to increases in the proliferation rate and the MMP of A375 cells, highlighting the relevance of mitoNEET as a regulator of the oxidative capacity of melanoma cells.

In summary, ISL induced apoptosis and mitochondrial dysfunction in A375 melanoma cells by inhibiting the expression of mitoNEET. The data provide evidence for a novel pathway in ISL-induced apoptosis of melanoma cells and highlight the unique role of mitoNEET as an essential molecule in mitochondrial injury and apoptosis.

## Data Availability

The data used to support the findings of this study are included in the article.

## Abbreviations

ISL: Isliquiritigenin
IMM: Mitochondrial inner membrane
OMM: Mitochondrial outer membrane
MOMP: Mitochondrial outer membrane permeabilization
ROS: Reactive oxygen species
CISD1: CDGSH iron sulfur domain 1
Casp.3: Caspase-3
NBT: Nitroblue tetrazolium
H_2_O_2_: Hydrogen peroxide
ROS: Reactive oxygen species
GSH: Reducted glutathione
GSSG: Oxidated glutathione
MTT: Methyl thiazolyl tetrazolium
ATP: Adenosine triphosphate
NAC: N-Acetyl-L-cysteine
BSO: Buthionine sulfoximine.

## Supplementary Materials

Supplementary Materials Figure S1: pLVX-CMV-MCS-T2A-Zsgreenn vector map. Figure S2: mitoNEET overexpression vector sequencing and matching result. Table S1: primers designed for mitoNEET and 3^∗^flag.

## References

[1] Norain A., Dadachova E. (2016). Targeted radionuclide therapy of melanoma. *Seminars in Nuclear Medicine*.

[2] Mahadevan A., Patel V. L., Dagoglu N. (2015). Radiation therapy in the management of malignant melanoma. *Oncology*.

[3] Gorayski P., Burmeister B., Foote M. (2015). Radiotherapy for cutaneous melanoma: current and future applications. *Future Oncology*.

[4] Schwarz M., Andrade-Navarro M. A., Gross A. (2007). Mitochondrial carriers and pores: key regulators of the mitochondrial apoptotic program?. *Apoptosis*.

[5] Liu C., Gong K., Mao X., Li W. (2011). Tetrandrine induces apoptosis by activating reactive oxygen species and repressing Akt activity in human hepatocellular carcinoma. *International Journal of Cancer*.

[6] Lane N., Martin W. (2010). The energetics of genome complexity. *Nature*.

[7] Wang C., Youle R. J. (2009). The role of mitochondria in apoptosis. *Annual Review of Genetics*.

[8] Kroemer G., Galluzzi L., Brenner C. (2007). Mitochondrial membrane permeabilization in cell death. *Physiological Reviews*.

[9] Vaux D. L. (2011). Apoptogenic factors released from mitochondria. *Biochimica et Biophysica Acta*.

[10] Halliwell B. (2011). Free radicals and antioxidants-quo vadis?. *Trends in Pharmacological Sciences*.

[11] Winterbourn C. C. (2015). Are free radicals involved in thiol-based redox signaling?. *Free Radical Biology & Medicine*.

[12] Chen Q., Vazquez E. J., Moghaddas S., Hoppel C. L., Lesnefsky E. J. (2003). Production of reactive oxygen species by mitochondria: central role of complex III. *The Journal of Biological Chemistry*.

[13] Liu Y., Fiskum G., Schubert D. (2002). Generation of reactive oxygen species by the mitochondrial electron transport chain. *Journal of Neurochemistry*.

[14] Turrens J. F. (2003). Mitochondrial formation of reactive oxygen species. *The Journal of Physiology*.

[15] Orrenius S., Gogvadze V., Zhivotovsky B. (2015). Calcium and mitochondria in the regulation of cell death. *Biochemical and Biophysical Research Communications*.

[16] Circu M. L., Aw T. Y. (2010). Reactive oxygen species, cellular redox systems, and apoptosis. *Free Radical Biology & Medicine*.

[17] Taminelli G. L., Sotomayor V., Valdivieso A. G., Teiber M. L., Marín M. C., Santa-Coloma T. A. (2008). CISD1 codifies a mitochondrial protein upregulated by the CFTR channel. *Biochemical and Biophysical Research Communications*.

[18] Wiley S. E., Andreyev A. Y., Divakaruni A. S. (2013). Wolfram syndrome protein, Miner1, regulates sulphydryl redox status, the unfolded protein response, and Ca^2+^ homeostasis. *EMBO Molecular Medicine*.

[19] Zuris J. A., Harir Y., Conlan A. R. (2011). Facile transfer of [2Fe-2S] clusters from the diabetes drug target mitoNEET to an apo-acceptor protein. *Proceedings of the National Academy of Sciences of the United States of America*.

[20] Simoneau J. A., Veerkamp J. H., Turcotte L. P., Kelley D. E. (1999). Markers of capacity to utilize fatty acids in human skeletal muscle: relation to insulin resistance and obesity and effects of weight loss. *The FASEB Journal*.

[21] Wiley S. E., Murphy A. N., Ross S. A., van der Geer P., Dixon J. E. (2007). MitoNEET is an iron-containing outer mitochondrial membrane protein that regulates oxidative capacity. *Proceedings of the National Academy of Sciences of the United States of America*.

[22] Kusminski C. M., Holland W. L., Sun K. (2012). MitoNEET-driven alterations in adipocyte mitochondrial activity reveal a crucial adaptive process that preserves insulin sensitivity in obesity. *Nature Medicine*.

[23] Sohn Y. S., Tamir S., Song L. (2013). NAF-1 and mitoNEET are central to human breast cancer proliferation by maintaining mitochondrial homeostasis and promoting tumor growth. *Proceedings of the National Academy of Sciences of the United States of America*.

[24] Chen X., Yang M., Hao W. (2016). Differentiation-inducing and anti-proliferative activities of isoliquiritigenin and all-trans-retinoic acid on B16F0 melanoma cells: mechanisms profiling by RNA-seq. *Gene*.

[25] Chen X., Zhang B., Yuan X. (2012). Isoliquiritigenin-induced differentiation in mouse melanoma B16F0 cell line. *Oxidative Medicine and Cellular Longevity*.

[26] Li D., Wang Z., Chen H. (2009). Isoliquiritigenin induces monocytic differentiation of HL-60 cells. *Free Radical Biology & Medicine*.

[27] Yuan X., Zhang B., Chen N. (2012). Isoliquiritigenin treatment induces apoptosis by increasing intracellular ROS levels in HeLa cells. *Journal of Asian Natural Products Research*.

[28] Wang Y., Ma J., Yan X. (2016). Isoliquiritigenin inhibits proliferation and induces apoptosis via alleviating hypoxia and reducing glycolysis in mouse melanoma B16F10 cells. *Recent Patents on Anti-Cancer Drug Discovery*.

[29] Quoilin C., Mouithys-Mickalad A., Lécart S., Fontaine-Aupart M. P., Hoebeke M. (2014). Evidence of oxidative stress and mitochondrial respiratory chain dysfunction in an *in vitro* model of sepsis-induced kidney injury. *Biochimica et Biophysica Acta (BBA) - Bioenergetics*.

[30] Ichikawa H., Wang X., Konishi T. (2003). Role of component herbs in antioxidant activity of Shengmai San — a traditional Chinese medicine formula preventing cerebral oxidative damage in rat. *The American Journal of Chinese Medicine*.

[31] Tian R., Xu S., Lei X., Jin W., Ye M., Zou H. (2005). Characterization of small-molecule-biomacromolecule interactions: from simple to complex. *TrAC Trends in Analytical Chemistry*.

[32] Peng F., Du Q., Peng C. (2015). A review: the pharmacology of isoliquiritigenin. *Phytotherapy Research*.

[33] Jung S. K., Lee M. H., Lim D. Y. (2014). Isoliquiritigenin induces apoptosis and inhibits xenograft tumor growth of human lung cancer cells by targeting both wild type and L858R/T790M mutant EGFR. *Journal of Biological Chemistry*.

[34] Kim D. H., Park J. E., Chae I. G., Park G., Lee S., Chun K. S. (2017). Isoliquiritigenin inhibits the proliferation of human renal carcinoma Caki cells through the ROS-mediated regulation of the Jak2/STAT3 pathway. *Oncology Reports*.

[35] Zhang B., Lai Y., Li Y. (2018). Antineoplastic activity of isoliquiritigenin, a chalcone compound, in androgen-independent human prostate cancer cells linked to G2/M cell cycle arrest and cell apoptosis. *European Journal of Pharmacology*.

[36] Vizetto-Duarte C., Custódio L., Gangadhar K. N. (2016). Isololiolide, a carotenoid metabolite isolated from the brown alga *Cystoseira tamariscifolia*, is cytotoxic and able to induce apoptosis in hepatocarcinoma cells through caspase-3 activation, decreased Bcl-2 levels, increased p53 expression and PARP cleavage. *Phytomedicine*.

[37] Jacquemin G., Margiotta D., Kasahara A. (2015). Granzyme B-induced mitochondrial ROS are required for apoptosis. *Cell Death and Differentiation*.

[38] Chen H., Zhang B., Yuan X. (2013). Isoliquiritigenin-induced effects on Nrf2 mediated antioxidant defence in the HL-60 cell monocytic differentiation. *Cell Biology International*.

[39] Tamir S., Paddock M. L., Darash-Yahana-Baram M. (2015). Structure-function analysis of NEET proteins uncovers their role as key regulators of iron and ROS homeostasis in health and disease. *Biochimica et Biophysica Acta (BBA) - Molecular Cell Research*.

[40] Shulga N., Pastorino J. G. (2014). Mitoneet mediates TNFα-induced necroptosis promoted by exposure to fructose and ethanol. *Journal of Cell Science*.

