# Supplementary material for: Isoliquiritigenin Induces Mitochondrial Dysfunction and Apoptosis by Inhibiting mitoNEET in a Reactive Oxygen Species-Dependent Manner in A375 Human Melanoma Cells
Source: Oxid Med Cell Longev. 2019 Jan 21;2019:9817576. doi: 10.1155/2019/9817576 (PMC6360568; doi:10.1155/2019/9817576)
Supplement: Supplementary Materials — Figure S1: pLVX-CMV-MCS-T2A-Zsgreenn vector map. Figure S2: mitoNEET overexpression vector sequencing and matching result. Table S1: primers designed for mitoNEET and 3∗flag. [file 9817576.f1.pdf]

Supplemental data

Figure S1. pLVX-CMV-MCS-T2A-Zsgreenn vector map.

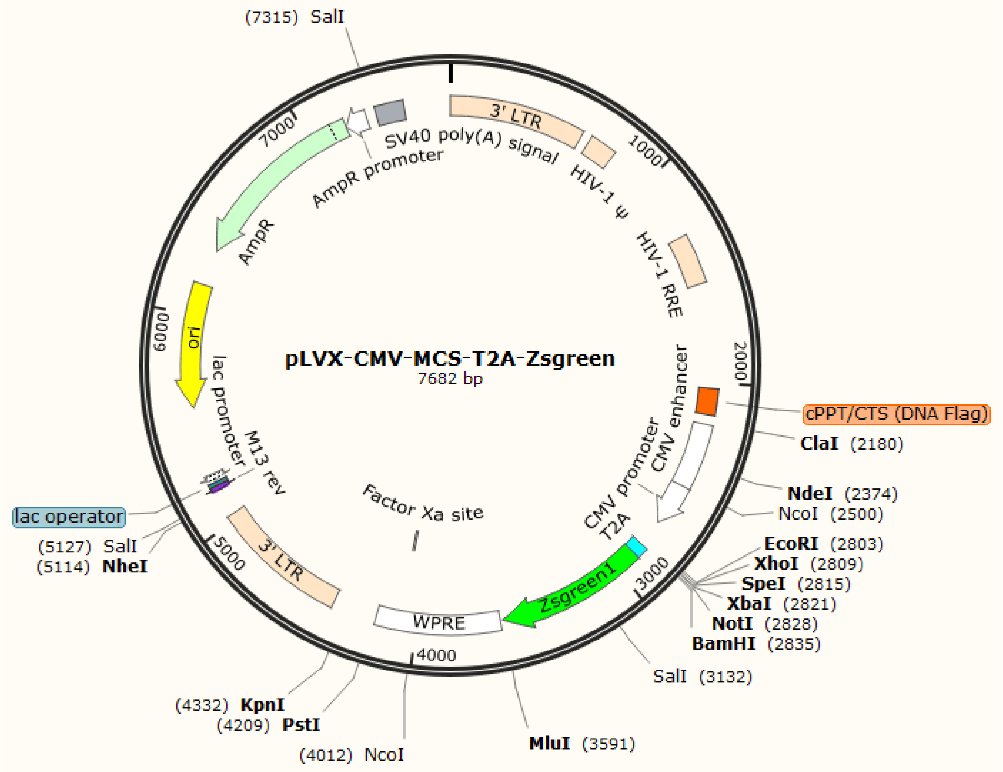

Figure S2. mitoNEET overexpression vector sequencing and matching result.

CCGATGGGGGGCATATAGCAGAGCTCGTTTAGTGACCGTCAGATCGCCTGGAGACGCCATCCACGCTG
TTTTGACCTCCATAGAAGACACCGACTTTACTAGAGGATCTATTTCCGGTGAATTCGCCACCATGAGTC
TGACTTCCAGTTCCAGCGTACGAGTTGAATGGATCGCAGCAGTTACCATTGCTGCTGGGACAGCTGCAA
TTGGTTATCTAGCTTACAAAAGATTTTATGTTAAAGATCATCGAAATAAAGCTATGATAAACCTTCACA
TCCAGAAAGACAACCCCAAGATAGTACATGCTTTTGACATGGAGGATTTGGGAGATAAAGCTGTGTACT
GCCGTTGTTGGAGGTCCAAAAAGTTCCCATTCTGTGATGGGGCTCACACAAAACATAACGAAGAGACTG
GAGACAATGTGGGCCCTCTGATCATCAAGAAAAAAGAAACTGACTACAAGGATGACGATGACAAGGATT
ACAAAGACGACGATGATAAGGACTATAAGGATGATGACGACAAAGGATCCGAGGGCAGAGGAAGTCTTC
TAACATGCGGTGACGTGGAGGAGAATCCCGGCCCTATGGCCCAGTCCAAGCACGGCCTGACCAAGGAGA
TGACCATGAAGTACCGCATGGAGGGCTGCGTGGACGGCCACAAGTTCGTGATCACCGGCGAGGGCATCG
GCTACCCCTTCAAGGGCAAGCAGGCCATCAACCTGTGCGTGGTGGAGGGCGGCCCCTTGCCCTTCGCCG
AGGACATCTTGTCCGCCGCCTTCATGTACGGCAACCGCGTGTTCACCGAGTACCCCCAGGACATCGTCG
ACTACTTCAAGAACTCCTGCCCCGCCGGCTACACCTGGGACCGCTCCTTCCTGTTCGAGGACGGCGCCG
TGTGCATCTGCAACGCCGACATCA

mitoNEET overexpression vector sequencing result. (objective sequence highlighted in yellow and 3*flag sequence highlighted in purple).

```
CISD1+3flag?          ----------------------------------------------------------------------
W24023_W1849      1   ccgatgggggggcatatagcagagctcgtttagtgaccgtcagatcgcctggagacgccatccacgctgt

CISD1+3flag?      1   -------------------------------------------------------------atgagtctg
W24023_W1849     71   tttgacctccatagaagacaccgactttactagaggatctatttccggtgaattcgccaccatgagtctg

CISD1+3flag?     10   acttccagttccagcgtacgagttgaatggatcgcagcagttaccattgctgctgggacagctgcaattg
W24023_W1849    141   acttccagttccagcgtacgagttgaatggatcgcagcagttaccattgctgctgggacagctgcaattg

CISD1+3flag?     80   gttatctagcttacaaaagattttatgttaaagatcatcgaaataaagctatgataaaccttcacatcca
W24023_W1849    211   gttatctagcttacaaaagattttatgttaaagatcatcgaaataaagctatgataaaccttcacatcca

CISD1+3flag?    150   gaaagacaaccccaagatagtacatgcttttgacatggaggatttgggagataaagctgtgtactgccgt
W24023_W1849    281   gaaagacaaccccaagatagtacatgcttttgacatggaggatttgggagataaagctgtgtactgccgt

CISD1+3flag?    220   tgttggaggtccaaaaagttcccattctgtgatggggctcacacaaaacataacgaagagactggagaca
W24023_W1849    351   tgttggaggtccaaaaagttcccattctgtgatggggctcacacaaaacataacgaagagactggagaca

CISD1+3flag?    290   atgtgggccctctgatcatcaagaaaaaagaaactgactacaaggatgacgatgacaaggattacaaaga
W24023_W1849    421   atgtgggccctctgatcatcaagaaaaaagaaactgactacaaggatgacgatgacaaggattacaaaga

CISD1+3flag?    360   cgacgatgataaggactataaggatgatgacgacaaa---------------------------------
W24023_W1849    491   cgacgatgataaggactataaggatgatgacgacaaaggatccgagggcagaggaagtcttctaacatgc

CISD1+3flag?          ----------------------------------------------------------------------
W24023_W1849    561   ggtgacgtggaggagaatcccggccctatggcccagtccaagcacggcctgaccaaggagatgaccatga

CISD1+3flag?          ----------------------------------------------------------------------
W24023_W1849    631   agtaccgcatggagggctgcgtggacggccacaagttcgtgatcaccggcgagggcatcggctacccctt
```

mitoNEET overexpression vector matching result. (green region showed objective sequence of mitoNEET overexpression vector matched mitoNEET fragment).

Table S1. Primers designed for mitoNEET and 3*flag.

h-CISD1-Eco/Bam-F	ggatctatttccggtgaattc gccacc ATGAGTCTGACTTCCAGT
h-CISD1-r	TCGTCATCCTTGTAGTCAGTTTCTTTTTCTTGATGAT
3flag（FNDC5）-f	GACTACAAGGATGACGATGA
3flag(FNDC5)-Eco/Bam-R	acttcctctgccctcggatcc TTTGTCGTCATCATCCT
